# Sub-MHz EMAR for Non-Contact Thickness Measurement: How Ultrasonic Wave Directivity Affects Accuracy

**DOI:** 10.3390/s25154746

**Published:** 2025-08-01

**Authors:** Alexander Siegl, David Auer, Bernhard Schweighofer, Andre Hochfellner, Gerald Klösch, Hannes Wegleiter

**Affiliations:** 1 Christian Doppler Laboratory for Measurement Systems for Harsh Operating Conditions, Graz University of Technology, Inffeldgasse 23/2, 8010 Graz, Austriabernhard.schweighofer@tugraz.at (B.S.); wegleiter@tugraz.at (H.W.); 2 voestalpine Stahl Donawitz GmbH, Kerpelystrasse 199, 8700 Leoben, Austria

**Keywords:** electromagnetic acoustic resonance, thickness gauging, electromagnetic acoustic transducer, EMAR limitations, EMAR simulation

## Abstract

Electromagnetic acoustic resonance (EMAR) is a well-established non-contact method for ultrasonic thickness measurement, typically operated at frequencies above 1 MHz using an electromagnetic acoustic transducer (EMAT). This study successfully extends EMAR into the sub-MHz range, allowing supply voltages below 60 V and thus offering safer and more cost-effective operation. Experiments were conducted on copper blocks approximately 20 mm thick, where a relative thickness accuracy of better than 0.2% is obtained. Regarding this result, the research identifies a critical design principle: Stable thickness resonances and subsequently accurate thickness measurement are achieved when the ratio of ultrasonic wavelength to EMAT track width (λ/w) falls below 1. This minimizes the excitation and interactions with structural eigenmodes, ensuring consistent measurement reliability. To support this, the study introduces a system-based model to simulate the EMAR method. The model provides detailed insights into how wave propagation affects the accuracy of EMAR measurements. Experimental results align well with the simulation outcome and confirm the feasibility of EMAR in the sub-MHz regime without compromising precision. These findings highlight the potential of low-voltage EMAR as a safer, cost-effective, and highly accurate approach for industrial ultrasonic thickness measurements.

## 1. Introduction

In the field of non-destructive testing, thickness gauging of solid materials is used to determine wall thickness or to assess material wear over time [1,2,3]. One approach of measuring the thickness is to generate and measure ultrasonic waves inside the specimen with ultrasonic transducers [4,5]. A common way to induce ultrasonic waves is by a piezoelectric transducer [6,7,8]. It is attached to the specimen with a couplant to achieve proper mechanical coupling between the transducer and the specimen. However, there are industrial applications where the surface of the specimen is rough or has a temperature higher than 100 °C [9]. Those harsh environments limit the use of a piezoelectric transducer. One possible solution is to use a laser as a transducer. However, lasers are costly and bulky, and therefore, challenging to install in actual measurement applications [10,11].

An alternative is provided by the so called electromagnetic acoustic transducer (EMAT) [12,13,14]. It is used to generate and measure ultrasonic bulk waves as well as surface or guided waves without contact to the sample [15,16,17,18]. An EMAT can be built in a compact and cost efficient way and is suitable to conduct ultrasonic thickness gauging for applications in harsh environments [19,20,21,22].

**Figure 1:**
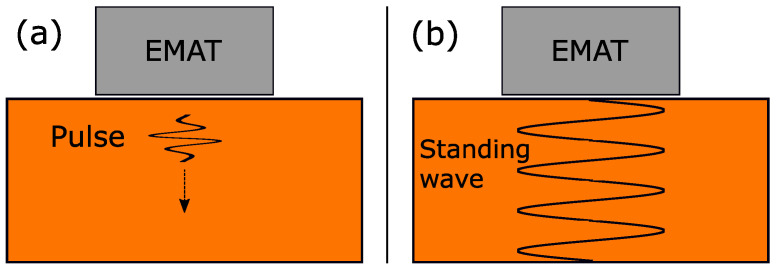
Illustration of EMAT operating methods. (**a**) Pulse-echo method: A short pulse is emitted and caught again by the EMAT. By measuring the ToF of the wave packet, the thickness is estimated. (**b**) EMAR method: A standing wave is implied beneath the EMAT, where the thickness can be estimated from the resonance frequency.

An EMAT can be operated in two ways. The first method, which is illustrated in Figure 1a is the pulse-echo method.

Here, the EMAT emits a short mechanical wave pulse into the specimen and catches it again after a certain time of flight (ToF), which is proportional to the thickness of the specimen [23,24,25]. Regarding the pulse-echo method, the poor coupling efficiency of the EMAT often makes it a challenging task to measure a signal with sufficient quality for the thickness estimation [26,27]. However, there is a further approach to the pulse-echo method to measure a specimen thickness with the EMAT, which is illustrated in Figure 1b. It is referred to as “electromagnetic acoustic resonance” (EMAR) [12,28]. The EMAR method implies that a standing wave along the thickness of the block is generated. This standing wave is the result of creating a mechanical resonance along the thickness, which leads to amplified mechanical movement inside the specimen. In this respect, EMAR produces a higher quality measurement signal compared to the pulse-echo method [12]. In order to excite the mechanical resonance, the excitation frequency has to match the resonance frequency

(1) $$f_{res,n}=n\frac{c}{2d}.$$

Here, *c* is the propagation velocity of the ultrasonic wave in the specimen, *d* is the thickness of the specimen and *n* is any positive integer.

Table 1 shows a brief review of related work regarding EMAR for thickness gauging, given the operating frequency, sample thickness, reported accuracy, and the highlights of each work.

As can be seen, EMAR is typically operated above 1 MHz. Thereby, it is not only used on thin plates or foils in the sub-millimeter to millimeter range where detection of wall thinning in the μm-region is achieved, but also on thicker walls or plates up to a thickness of 10 mm. Regarding the measurement time, EMAR measurements are also fast, e.g., Dixon et al. state real-time online monitoring is possible with their approach by only one single shot measurement; however, measurement accuracy decreases as plate thickness increases [29]. While Table 1 is focused on non-contact EMAR studies, it is acknowledged that other approaches also utilize thickness resonances. Notably, Heinlein et al. [3] demonstrated thickness gauging using guided waves with sub-MHz cut-off frequencies, which coincide with the thickness resonance frequencies exploited in the EMAR approach. Although their configuration involves spatially separated piezoelectric transducers, the underlying resonance condition remains the same. For completeness of the literature review, EMAR is not only used for thickness gauging, but also in the area of material characterization [28,30] and thin layer detection [31,32].

**Table 1:** Literature review on non-contact EMAR-based thickness gauging.

Author	Operating Frequency in MHz	Sample Thickness	Accuracy	Highlights
Kawashima et al. [33]	Up to 150	20 μm to 1mm	N/A	High frequency
Hobbis et al. [34]	>5	0.28 mm to 2.8 mm	0.08 μm (max. std)	Moving specimen
Dixon et al. [29]	>5	100 μm to 500 μm	0.2% (relative)	Rapid ‘single shot’ measurement
Chen et al. [35]	>1	0.5 mm to 3 mm	<4% (relative)	Laser/EMAT configuration
Li et al. [36]	>2	up to 8 mm	<2.5% (relative)	Sloped specimen
Yusa et al. [37]	1 to 4	up to 10 mm	±0.2 mm (absolute)	Probabilistic evaluation
Cai et al. [38]	2 to 6	up to 10 mm	0.9% (relative)	Specimen thickness step change
This work	0.04 to 1	20mm	<0.2% (relative)	Wave directivity impact on accuracy

As seen in Table 1, the published results for thickness gauging are obtained by operating the EMAR method at frequencies in the MHz range, typically higher than 1 MHz. The selected operating frequency depends, among other things, on the thickness of the specimen according to Equation (1). A corresponding brief numerical example to obtain the first possible resonance frequency of a copper sample with different thicknesses is shown in Table 2.

As can be seen, the first resonance frequency of a 1 mm thick copper sheet would be around 1.15 MHz. For the 20 mm thick copper sample, the first resonance frequency where EMAR theoretically works is around 58 kHz. Interestingly, even in related work where the samples are thicker (e.g., 10 mm), EMAR is still operated in the MHz frequency range, although theoretical operation below 1 MHz would be possible. Operating EMAR below 1 MHz offers additional advantages in terms of its applicability:

- Reduced supply voltage: As the EMAT consists of a coil, the overall impedance, and therefore, the required excitation voltage, is lower when the operation frequency is decreased. Indeed, as will be demonstrated in this work, EMAR is successfully operated at voltages below 60 V.
- Improved human and electrical safety: The lower operating voltage minimizes electrical hazards to personnel, eases compliance with safety regulations, and facilitates adaptation to standards for use in explosion-risk environments.
- Lower attenuation in the material: Depending on the ultrasonic frequency, the wave is naturally attenuated due to the material properties. In general, the attenuation in metals is lower at low frequencies and increases at higher frequencies [40]. That fact also promotes operation in the sub-MHz range.

In general, lower excitation frequencies make a possible realization of EMAR operation more comfortable in terms of necessary supply voltage and safety guidelines. Even though conventional high-frequency techniques may still be technically feasible, the practical considerations listed above illustrate why research into EMAR operation in the sub-MHz range is relevant for certain industrial applications.

Hence, this work provides a complementary study of the EMAR method when used at frequencies below 1 MHz. It addresses and analyzes the challenges and limitations of performing EMAR at lower frequencies compared with related work. As will be shown, the applicability of the EMAR method for thickness gauging encounters problems when used in the sub-MHz range. This work explains why these problems occur and identifies the conditions that must be met to successfully perform EMAR in this frequency range. In this regard, it is demonstrated that EMAR operation for thickness measurement in the sub-MHz range also achieves a relative accuracy better than 0.2 %.

For this work, copper blocks with a thickness of around 20 mm were selected as test specimens. As shown in Table 2, this thickness enables EMAR operation in the sub-MHz range. EMAR is then fundamentally investigated to estimate the thickness of the specimens based on the individual resonance frequencies.

The paper is structured as follows: In Section 2, the EMAT working principle is addressed by means of a certain EMAT design, and the EMAR method is explained in detail. Furthermore, in this section, the test specimens used for the experiments are introduced. Section 3 introduces the simulation framework for the EMAR study. Hereby, a system-based approach for simulating EMAR is presented. The corresponding simulation results are shown in Section 4. The actual measurement setup for the EMAR validation is presented in Section 5 and the measurement results are shown in Section 6. The thickness estimation based on the measurement results is presented in Section 7. Section 8 summarizes and discusses the results of this study.

## 2. Materials and Methods

This section presents the EMAT used in this work, explains the EMAR method and introduces the samples used in the experiments.

### 2.1. EMAT

The same EMAT is used for the ultrasonic wave excitation and detection without mechanical contact to the specimen. The EMAT working principle is well known and has been used in multiple applications [41,42,43]. There are three possible effects that can be used by an EMAT to generate mechanical waves in a specimen [12]:

- Magnetization
- Magnetostriction
- Lorentz forces

As shown later in this section, the specimens under test in this work are copper blocks, which are conductive materials and have a relative permeability of one. Hence, only the Lorentz force generation is present, and consequently, the other effects are negligible.

Lorentz forces are generated according to [12]

(2) $$\begin{array}{c} \vec{f_{L}}=\vec{J}\times \vec{B_{0}}. \end{array}$$

Equation (2) states that the Lorentz force density and its direction $\vec{f_{L}}$ are calculated by the cross product of the current density $\vec{J}$ in the specimen and the static magnetic flux density $\vec{B_{0}}$. To generate a current density $\vec{J}$ inside the specimen, eddy currents can be used. In terms of an EMAT, this is achieved by applying a time-varying current through a coil. The coil is typically manufactured on a printed circuit board (PCB). The current through the coil generates a time-varying magnetic field, which induces the eddy current spatially beneath the coil area. The second requirement is a static magnetic flux density $\vec{B_{0}}$, typically provided by a set of permanent magnets.

In order to receive the ultrasonic wave, the EMAT is considered a velocity sensor. The ultrasonic wave to be received, mechanically excites the particles inside the material and forces them to move with the velocity $\vec{v}$. In the presence of a static magnetic flux density, a time-varying current density [12]

(3) $$\begin{array}{c} \vec{J_{m}}=\sigma \left(\vec{v}\times \vec{B_{0}}\right) \end{array}$$

is generated inside the specimen with its electrical conductivity σ. This current density further generates a magnetic field, which induces a proportional voltage inside the EMAT coil. Its magnitude is proportional to the velocity $\vec{v}$ of the ultrasonic movements inside the specimen.

#### EMAT Design

**Figure 2:**
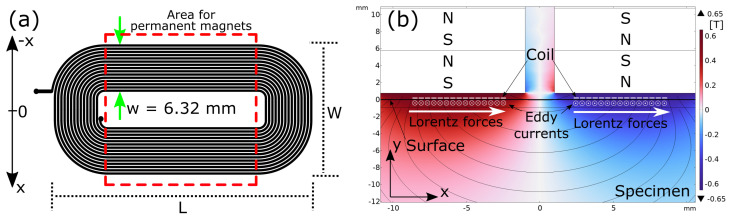
(**a**) Top view of racetrack coil configuration used for the EMAT. Each track spans a width of 6.32 mm. The red dotted square marks the area where the magnets are positioned. (**b**) 2D-Illustration of Lorentz force generation. A cross-section of the EMAT, including the racetrack coil and magnets on top, which provide the constant magnetic flux density, is shown. The color indicates the magnetic flux density $\vec{B}$ in y-direction.

Depending on the EMAT design, the EMAT is capable of generating and receiving certain wave types. According to [12], when using EMAR for thickness measurement, shear waves should be used. To generate shear waves within the material, a so-called racetrack EMAT is used [28]. It consists of a printed racetrack coil and a set of permanent magnets above the coil. A top view of the printed racetrack coil schematic is shown in Figure 2a.

To keep the EMAT handy for convenient use in the experiments, it has a length of L = 35 mm and a width W = 18 mm. Given these geometries and the design specifications of the PCB manufacturer, the EMAT ended up with N = 15 turns. They are spaced by 130 μm. In total, one track spans a width of w = 6.32 mm. This width is considered the track width. Both tracks are separated by a distance of 4 mm to primarily excite Lorentz forces parallel to the surface and thus enhance shear wave excitation. The red dashed area marks the area where the permanent magnets are put. The eddy currents and resulting Lorentz forces are generated within this area.

A cross-section of the EMAT with its permanent magnets, corresponding magnetic flux density field lines, eddy currents, and resulting Lorentz forces is shown in Figure 2b. As can be seen, the magnets are magnetized in different directions. The corresponding magnetic field lines in the vicinity of the coil are mainly normal to the surface of the specimen. Given the racetrack coil, the induced eddy currents directly beneath the surface point in opposite directions, and thus the resulting Lorentz forces will consequently point in the x-direction, and shear forces are generated. The magnetic field lines in the gap between the two tracks are mainly parallel to the surface and would also excite longitudinal waves. However, there are hardly any eddy currents induced in this gap. Therefore, the excitation of a possible longitudinal wave is weak compared with the shear wave excitation.

As the Racetrack EMAT is introduced for thickness measurement using EMAR, the next section briefly recaps the working principle of EMAR.

### 2.2. EMAR Working Principle

**Figure 3:**
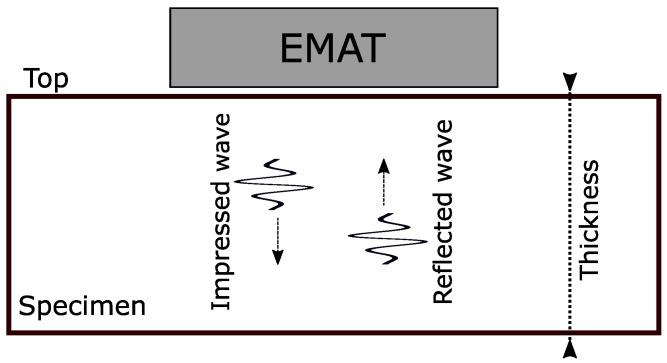
Depiction of EMAR working principle. The EMAT impresses a wave along the thickness of the specimen, which is reflected back to the top. When the timing is exact, the reflected wave is constructively interfering with another impressed wave from the EMAT.

The EMAR method assumes a standing wave along the thickness of the sample [12]. This standing wave is formed by a generated wave traveling along the thickness and is constantly reflected at the top and bottom of the specimen. For this measurement method, the EMAT is suitable as it generates the wave without mechanical contact to the specimen, and therefore, the reflection of the wave is not influenced by the transducer. Figure 3 shows an illustration of the theoretical reflection principle of ultrasonic waves. In resonance mode, the EMAT excites another wave just at the same time the first reflected wave arrives at the upper boundary.

If the timing is exact, the reflected wave from the bottom and the newly generated wave constructively interfere and form a higher resulting amplitude [12]. By repeating this procedure multiple times, the resulting ultrasonic wave amplitude increases further. The time points at which the wave has to be excited are a function of the ultrasonic wave speed inside the material, as well as the thickness of the block. The time it takes the wave to return to the upper surface is given by

(4) $$\begin{array}{c} t_{\mathrm{return}}=\frac{2d}{c}, \end{array}$$

with *d* the thickness of the block and *c* the ultrasonic velocity in the given material. If the excitation is repeated at the same time interval over and over again, a repetition frequency can be defined, and if the resonance condition is met, the amplitudes of each pulse add constructively, and therefore, the mechanical thickness resonance frequency can be defined. By further investigation, it turns out that each integer multiple of the resonance frequency satisfies the resonance condition, and thus, there is an infinite number of theoretical resonance frequencies given by

(5) $$\begin{array}{c} f_{\mathrm{res},n}=n\frac{1}{t_{\mathrm{return}}}=n\frac{c}{2d}, \end{array}$$

where *n* is any positive integer. If the ultrasonic wave speed is known and the resonance frequency is measured, then this method allows for determining the thickness of the block as

(6) $$\begin{array}{c} d=n\frac{c}{2f_{\mathrm{res},\mathrm{meas}}}. \end{array}$$

Instead of a pulse train, a continuous sine wave, which is often simpler to generate, can be used to excite the wave inside the specimen. Here, the resonance condition is met if the frequency of the sinusoidal excitation signal is equal to any of the resonance frequencies.

### 2.3. Test Samples

**Figure 4:**
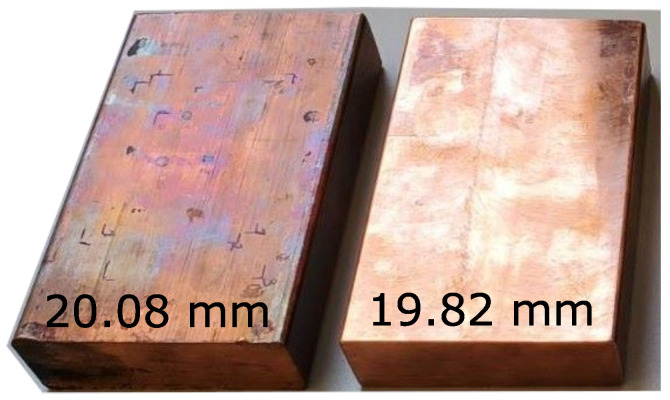
Test specimens used in this work. Two copper blocks are taken for the study. One has a thickness of 20.08 mm and the other one 19.82 mm, respectively. Those thickness values were determined with a caliper ( 10 μm resolution).

The test samples in this work are two copper blocks with a length of 100 mm and a width of 60 mm, but with different thicknesses. Figure 4 depicts the two specimens that are later used in the experiments.

One has a thickness of 20.08 mm and the other has a thickness of 19.82 mm. These thickness values are measured with a caliper providing a resolution of 10 μm and are considered as true thickness values throughout the work.

In order to operate EMAR on these blocks, one has to compute the shear wave speed for the material. When the mechanical properties of the copper are known, the shear wave velocity $c_{S}$ is computed by

(7) $$c_{S}=\sqrt{\frac{E}{2\rho (1+\nu )}}$$

Here, *E* is the Young’s Modulus, ρ is the density of the material and ν is referred to as Poisson’s ratio. The mechanical parameters for copper vary in a certain range depending on the copper composition and its processing. Table 3 gives an overview of the parameter range found in the literature [44,45] and our choice of the parameters for the EMAR validation.

As can be seen later in the measurements, the best thickness estimation is obtained when choosing the shear wave speed $c_{s}=2401.92\frac{m}{s}$. Given Equation (7), this shear wave speed is obtained when choosing the copper material parameters as shown in Table 3. This wave speed value serves as the first estimation for the computation of the resonance frequencies in the copper blocks. For this first estimation, an exemplary copper sample with the chosen mechanical parameters and thickness of 20 mm is assumed. According to Equation (5), the resonance frequencies are derived as

(8) $$f_{res,n}=n\;\cdot \;60.05\;\mathrm{kHz},$$

with *n* being any positive integer. Given Equation (8), the first 16 resonance frequencies are lower than 1 MHz, which now allows for this fundamental study of EMAR being operated below 1 MHz.

## 3. Simulation Setup

**Figure 5:**
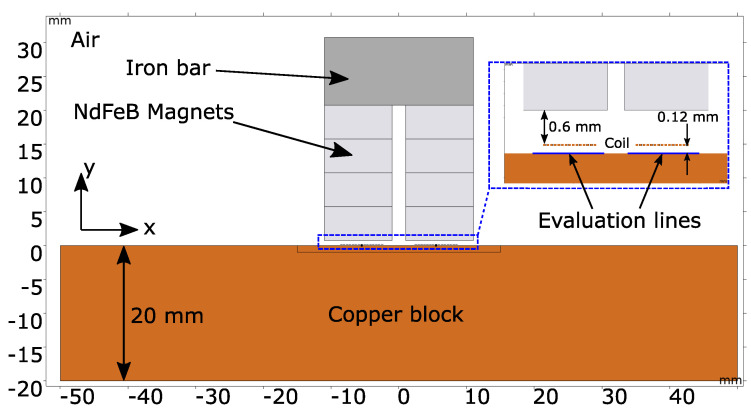
2D-FE model. The EMAT is centered on top of the 20 mm copper sample. It consists of the racetrack coil and stacked NdFeB magnets magnetically shorted on top with an iron bar. The EMAT is lifted off by 0.12 mm from the copper surface. The surrounding domain is air.

In order to verify the EMAR with respect to the thickness estimation of a copper block, a 2D simulation model is set up in COMSOL Multiphysics (Version 6.1). The finite element (FE) model is shown in Figure 5.

As can be seen, it represents a cross-section of the EMAT on top of the copper block. The modeled racetrack EMAT consists of 15 turns, where each coil segment is 0.3 mm wide and has a height of 35 μm. On top of the coil, at a distance of 0.6 mm, which corresponds to the thickness of the PCB, the permanent magnets are placed. They are sintered neodymium (NdFeB-N42) magnets which have a remanent flux density of 1.31 T and provide the static magnetic field for the ultrasonic wave generation. The EMAT is lifted from the copper sample by 0.12 mm. This air gap height is later applied in the experiment as well by putting a single layer of tape at the border of the PCB. The air gap is mandatory to avoid any mechanical interaction between the EMAT and the block, as this would affect the standing resonance wave along the thickness. The copper block is modeled as a rectangle with a thickness of exactly 20 mm and a length of 100 mm. The chosen material parameters from Table 3 are assigned. The EMAT is centered on top of the copper sample. The EMAT and the copper block are surrounded by an air domain. Using COMSOL, the static magnetic field, the eddy current induction, the generation of the Lorentz forces, as well as the mechanical pulse propagation are simulated.

Furthermore, the mesh size has to be chosen properly, especially inside the copper domain, where the wave propagation takes place. For the EMAR verification, frequencies up to 1 MHz are considered. At this frequency, the physical wavelength of the ultrasonic wave is $\lambda =c_{S}/1\;\mathrm{MHz}=2.4\mathrm{mm}$. According to modeling recommendations, the mesh size should be at most $\frac{\lambda }{10}$ to properly simulate the wave propagation. Thus, the mesh inside the copper sample is set to a maximum size of 240 μm.

Two evaluation lines are placed directly on the upper edge of the copper surface and beneath the left and right coil tracks. Along the lines, the velocity component in the x-direction is integrated and then divided by the length of the line to obtain the mean velocity in the x-direction at every time instance. This mean velocity below the coil tracks is responsible for generating a proportional voltage inside the EMAT coil as shown in Equation (3).

### System Identification

The model introduced above is used to verify the EMAR method from 40 kHz to 1 MHz, as well as to exemplarily illustrate the wave propagation in the copper specimen. One way to validate the EMAR simulation is to impress a sinusoidal pulse train or continuous excitation current through the coil for a certain amount of time and then perform line evaluations of the velocity in the x-direction for the mechanical response. This can then be conducted for each excitation frequency. However, this approach is expensive in terms of simulation time and evaluation.

Another approach is to characterize the EMAT with the copper block as a linear system with its impulse response h(t). The system input x(t) is the current through the coil and the output y(t) is the sum of the integrated velocity in the x-direction of the two evaluation lines. The velocity in this direction induces a proportional voltage in the EMAT, which is measured later on in the experiments. In order to compute the impulse response h(t), a step response is used. Hereby, a current step at the input is applied, and the output in the time domain is evaluated. The simulation time for the system step response is 250 μs in steps of 1 ns. As will be shown later, this simulation time is sufficient for the validation of the EMAR simulation.

Once the step response is acquired, it is differentiated in the time domain to obtain the impulse response h(t) of the system. The impulse response includes the mechanical description of the system over time and thus holds the resonance condition used for EMAR.

Given the simulated impulse response from the simulation, one can then apply any time signal at the input to compute the output of the system by the convolution of the input with the impulse response as

(9) y(t)=h(t)∗x(t).

The convolution operation is fast and can be performed in a program like Python or MATLAB (R2022b). Hence, with this approach, only one FEM-based simulation is required to generate the step response, and the EMAR method is verified within post-processing of the simulation. For EMAR, a sinusoidal pulse train with frequencies from 40 kHz to 1 MHz, in steps of $f_{res,1}/5$, are applied for 125 μs as input and the output for every excitation frequency is transferred into the frequency domain for generating the EMAR spectrum.

## 4. Simulation Results

**Figure 6:**
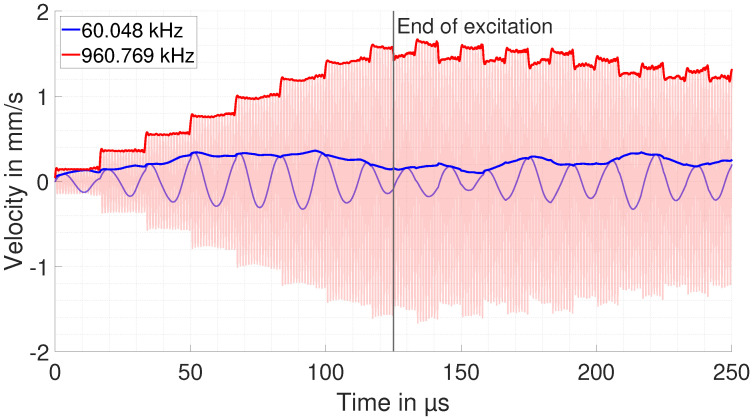
Convolution output at the first and 16th resonance, representing the amplitude of the copper particle velocity within the ultrasonic wave. Although the resonance condition is met, no significant constructive interference is seen for the first resonance frequency. For the 16th resonance frequency, the output adds up nicely every time the wave returns from the bottom surface until the end of the excitation.

As described above, the main simulation result from the COMSOL domain is the step response of the system h(t). It is convoluted with a sinusoidal excitation with frequencies from 40 kHz to 1 MHz. The output of this convolution represents the amplitude of the copper particle velocity within the ultrasonic wave. An exemplary result of two convolutions at the first and 16th resonance frequency with a sinusoidal current (amplitude of 1 A) is shown in Figure 6.

**Figure 7:**
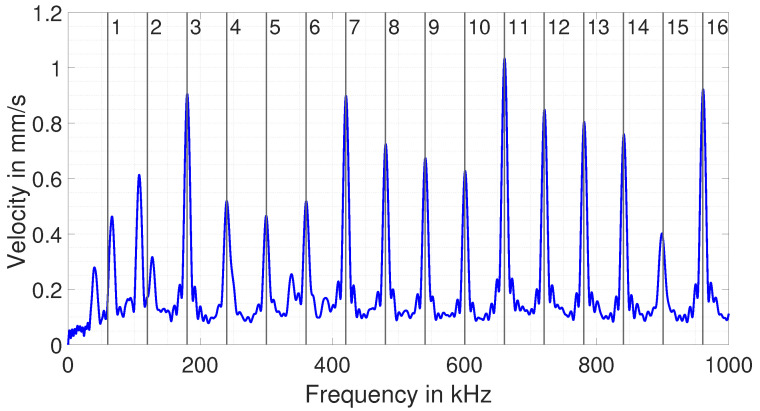
Computed EMAR resonances given by the simulated step response and further computation.

Although both frequencies theoretically meet the EMAR condition, only the excitation at the 16th resonance frequency adds up nicely during excitation time and then remains high for the rest of the time. In the case of the first resonance frequency, the signal does not add up much, and thus, after the end of excitation, it is lower by a factor of 8 compared with the higher frequency. The reason for this is addressed later on in this section. In order to compute the EMAR spectrum from the convolution, the part of the signal from 125 μs to 250 μs (time range in which the excitation is already over) is transferred into the frequency domain for every excitation frequency and one obtains the desired EMAR spectrum. The spectrum from the simulation is shown in Figure 7.

As can be seen, the spectrum shows that the simulated resonances match the calculated frequencies of Equation (8) for n≥3. These resonances confirm the system-based approach for simulating EMAR. However, it is also seen that the first and second resonance frequencies do not fit with the theoretical frequencies. This is actually the result of the velocity not adding up during excitation, as shown in Figure 6. Here, EMAR runs into problems when trying to use it for thickness estimation. And the reason for this problem is the weak directivity of the impressed wave towards the bottom of the block, as shown in the next section.

### Directivity

**Figure 8:**
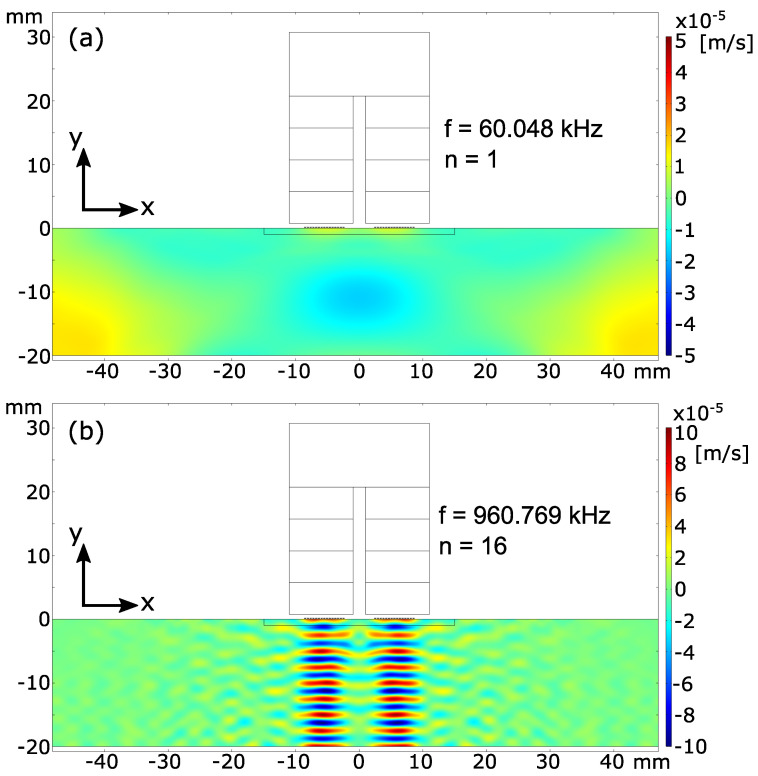
Simulated standing wave motion inside the copper block in terms of velocity in x-direction at 15.85 μs. (**a**) First resonance frequency. (**b**) 16th resonance frequency.

To analyze the directivity, the simulated wave motion of the propagating wave is illustrated at a certain point in time. Hereby, the first and 16th resonance frequency are examined. Figure 8 shows the simulated standing wave motion (x-velocity) along the block thickness for the two frequencies at 15.85 μs. This point in time is chosen as the wave was already reflected back once from the bottom of the block and is propagating towards the top again.

It can clearly be seen that the wave in Figure 8a does not propagate nicely below the EMAT. Indeed, a strong movement is also seen on the side walls, indicating the entire domain is mechanically excited. For this frequency, the impressed wave does not stay spatially beneath the EMAT and thus no constructive interference builds up. A completely different behavior is seen in Figure 8b. Here, the wave movement is more focused towards the bottom and stays spatially beneath the EMAT. In this case, the constructive interference takes place and a standing wave along the thickness builds up.

The simulation results already clearly show that the impressed wave hardly remains spatially below the EMAT when attempting to perform EMAR at very low frequencies. Instead, it mechanically excites the entire test specimen. As a result, the so-called eigenmodes of the blocks are excited, which leads to a shift in the actual thickness resonance frequency and generates additional resonance frequencies. This is especially seen for frequencies < 150 kHz as shown in Figure 7. These resonances are detected by the EMAT as well and thus falsify a potential thickness evaluation.

In order to verify this directivity behavior seen in the simulation, experiments in a laboratory environment are performed and compared with the outcome of the simulation.

## 5. Measurement Setup and Signal Processing

**Figure 9:**
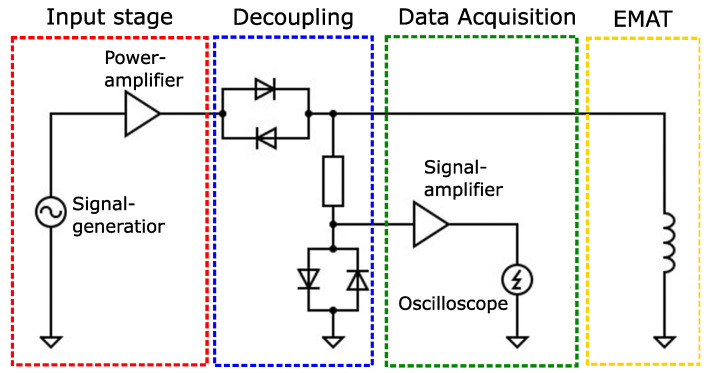
Schematic illustration of the measurement setup. The input stage provides the high current for driving the EMAT. The commonly used decoupling network allows for the EMAT to be used as transmitter and receiver. The received EMAT voltage is amplified and stored within the data acquisition block. The last part is considered the EMAT itself.

This section describes the measurement setup for thickness measurement with EMAR and the signal processing. The aim of the measurement experiments is to examine the applicability of the EMAR method for estimating the specimen thickness on the basis of the individual resonance frequencies. Furthermore, the results are compared with the outcome of the simulation. An illustrative overview of the measurement setup is seen in Figure 9.

The setup consists of four parts. The first part is the input stage, consisting of a signal generator and a power amplifier. The input stage is responsible for providing the required signal form and current to drive the EMAT. The second part is the decoupling network. As shown, it decouples the excitation signal from the input to the signal amplifier. In this way, the EMAT can be used as a transmitter and receiver. The third part is the data acquisition, where the EMAT signal generated from the ultrasonic wave is amplified and then measured by a USB-oscilloscope. The last part is the EMAT itself, acting as a generator for the excitation of the ultrasonic wave and subsequently as a receiver to measure the ultrasonic wave movement.

**Figure 10:**
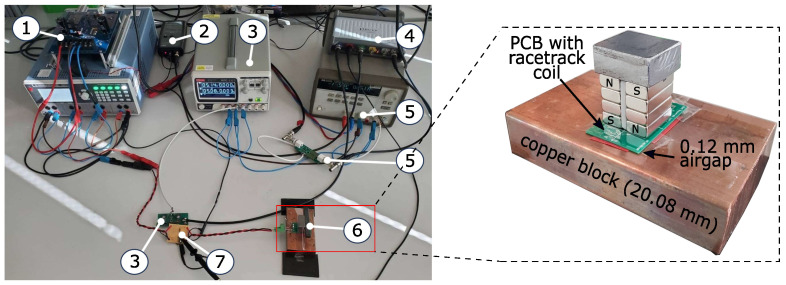
Measurement setup used in laboratory environment. 1: Power amplifier with power supply; 2: Current probe; 3: Amplifier for the measured EMAT signal and power supply; 4: Picoscope (Signal generator and data acquisition); 5: Pre-amplifier and power supply; 6: EMAT on copper block; 7: Decoupling network.

A picture of the actual setup used in the laboratory environment and the EMAT on the copper block is shown in Figure 10.

**Figure 11:**
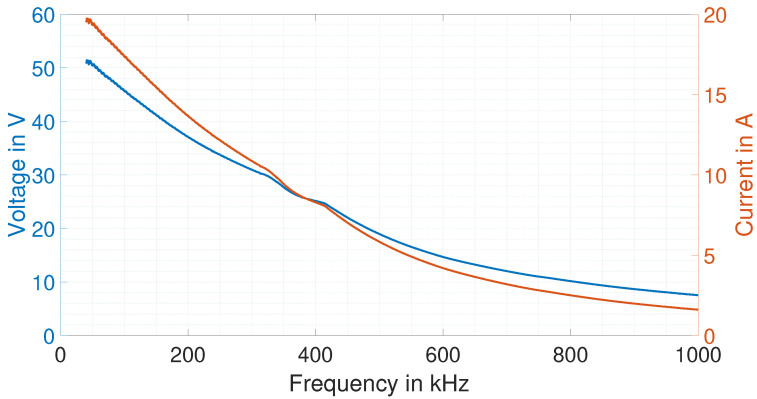
Measured current amplitude during the EMAT excitation and corresponding calculated voltage provided by the power amplifier.

For the power amplifier, the APEX PA05 (Apex Microtechnology, Tucson, AZ 85741, USA) is used. This amplifier can drive output currents up to 20 A. The decoupling network is built on a PCB, and the signal amplifier is built as a non-inverting amplifier with a gain of 100. The EMAT is placed at the center of the copper block and lifted 0.12 mm from the surface of the block by means of a tape at the border of the PCB. This air gap height is the same as in the simulation. Given the EMAT on the copper block, an ohmic part of R=2.6 Ω and an inductive part of *L* = 0.62 μH of the racetrack coil were measured. The overall absolute value of the coil impedance is

(10) $$|\underline{Z(f)}|=\sqrt{R^{2}+{\left(2\pi fL\right)}^{2}},$$

with *f* as the excitation frequency. The sinusoidal current amplitude during the excitation phase throughout the experiments is measured for every excitation frequency, and the voltage amplitude at each frequency is computed with the absolute value of the impedance $|\underline{Z(f)}|$. Figure 11 depicts the measured current through the EMAT and the corresponding voltage over the excitation frequencies.

As shown and mentioned in the introduction, the EMAR operation is performed with supply voltages lower than 60 V. This lower voltage is advantageous in practice, as it simplifies the requirements for power electronics and insulation and increases operational safety, while still providing a substantial amount of current through the EMAT for the successful excitation of thickness resonances as will be shown later. As excitation frequency increases, the voltage decreases due to limitations of the power amplifier. Consequently, the current also decreases.

### Measurement Procedure

**Figure 12:**
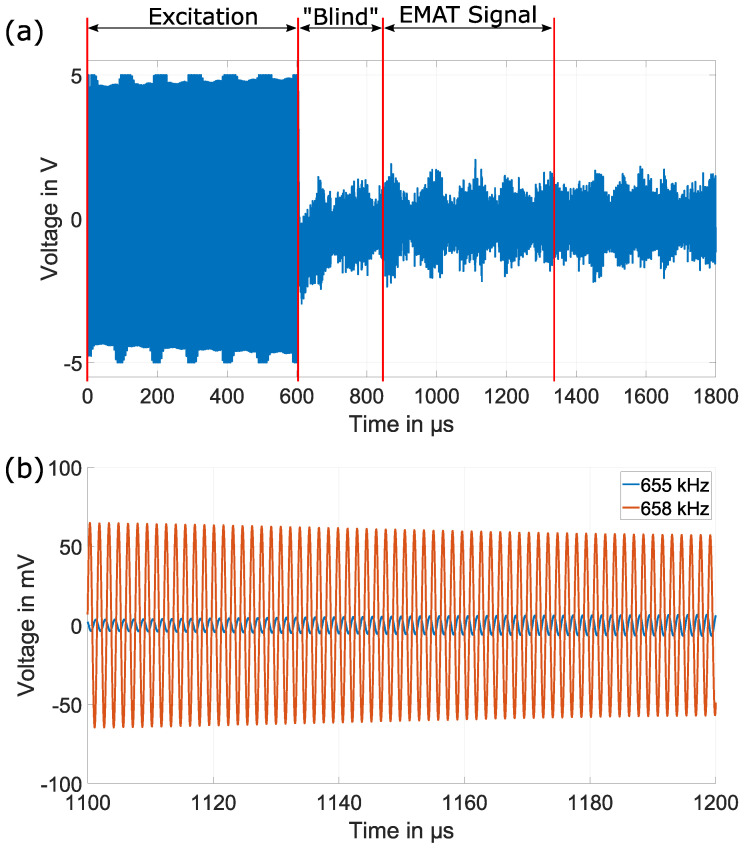
(**a**) Exemplary unfiltered output from the signal amplifier at excitation frequency of 658 kHz. (**b**) Zoom into the bandpass filtered EMAT time signals for excitation frequencies of 655 kHz and 658 kHz within the 500 μs evaluation window.

The EMAR method is verified for the frequency range of 40 kHz to 1 MHz in steps of 250 Hz. Within this frequency range, the first 16 resonance frequencies are expected to be excited within the specimen as follows. By means of the signal generator, a sine train with the desired excitation frequency is provided to the power amplifier, which drives the excitation current through the EMAT. The duration of the excitation is set to 600 μs. Depending on the excitation frequency, 24 to 600 pulses within the sine train are applied at the input. Figure 12a exemplary depicts the unfiltered output of the signal amplifier at an excitation frequency of 658 kHz for the described measurement procedure. The sampling rate of the Picoscope is set to 20 MHz.

During excitation, the output of the amplifier is driven into saturation. After the excitation, there is a “blind” window of about 250 μs where no measurements are possible due to necessary signal settling. After 850 μs, the EMAT signal is measured. As can be seen, the EMAT signal is quite noisy, but additional signal post-processing, including filtering operations, will help to extract the necessary information for the EMAR operation.

For the post-processing, the measured EMAT signal is truncated to a length of 500 μs. With regard to the choice of this time window, various lengths were investigated, and the window position was also shifted. However, the previously given parameters (starting time and length) provide the best results in terms of signal amplitude. With the length of 500 μs, a resolution of 2 kHz is achieved in the frequency domain. As will be seen later, this resolution is sufficient to correctly resolve and determine the position of the resonance peaks. The signal within this time window is additionally bandpass filtered at the given excitation frequency. The filter has a bandwidth of 10 kHz. A zoom into the bandpass filtered EMAT time signals between 1100 μs and 1200 μs for exemplary frequencies 655 kHz and 658 kHz is shown in Figure 12b. These signals are measurements from the 20.08 mm copper block. As can be seen, the amplitude of the signal at 658 kHz is roughly 10 times or 20 dB higher compared with the signal at 655 kHz. That is because at 658 kHz, the 11th resonance frequency is mechanically excited within the copper block, as will be shown later. Within this time window, so-called coherent sampling [46] is performed by cutting out an integer number of periods for the given excitation frequency. From this sequence, the Fourier transform is computed, and the voltage value at the excitation frequency is extracted over the entire range of excitation frequencies and plotted for the contribution to the final EMAR spectrum.

## 6. Measurement Results

As described above, the aim of the measurement experiments and the corresponding results is to examine the applicability of the EMAR method for estimating the specimen thickness on the basis of the individual resonance frequencies of the EMAR spectrum.

**Figure 13:**
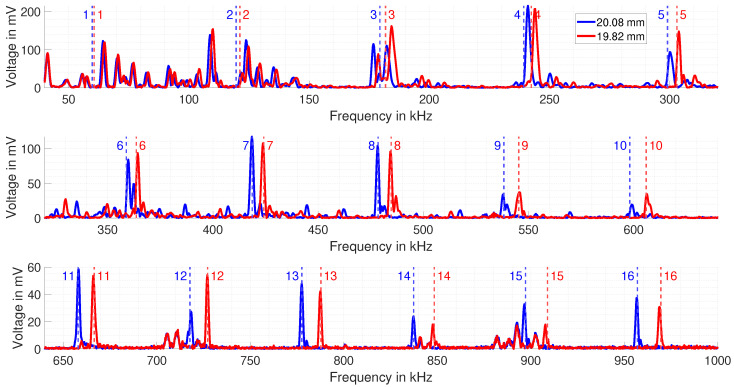
Measured EMAR spectrum in the frequency range from 40 kHz up to 1 MHz. In blue, the spectrum for the 20.08 mm block is shown and in red the spectrum for the 19.82 mm block is depicted. Furthermore, the predicted resonance frequencies for each thickness are included.

The measured EMAR spectrum for the test samples is shown in Figure 13.

In blue, the spectrum of the 20.08 mm copper block is plotted, and in red, the spectrum of the 19.82 mm block is shown. The spectrum is divided into three single spectra for better illustration. In the first row, the frequencies from 40 kHz to 320 kHz are plotted. Within this frequency range, the first five resonance frequencies are contained. The second row, ranging from 320.25 kHz to 640 kHz, includes the resonance frequencies number 6 to 10. The third row contains the last six resonance frequencies from number 11 to 16. Each row also contains the predicted resonant frequencies for the 20.08 mm thick copper block and for the 19.82 mm block. The predicted resonances are numbered vertical dashed lines in the corresponding color for each specimen. The number marks the *n*th predicted resonance frequency. What can already be seen is that the maximum peak height of the measured resonances decreases for increasing frequency (Please note the different vertical axis scaling). This is due to the limitations of the power amplifier, leading to decreasing current at higher frequencies.

When analyzing each row shown in the measured spectrum in Figure 13, one can see in the first row that the predicted resonance frequencies poorly match the actual measured resonance frequencies. Especially for the resonance frequencies 1 to 3, multiple peaks around the predicted resonance frequencies are observed. These peaks arise from the fact that the directivity of the ultrasonic wave towards the bottom of the block is weak when trying to generate a standing mechanical wave along the thickness of the block, as shown in Figure 8. Thus, these peaks from 1 to 3 are actual mechanical excited resonances from the eigenmodes of the block and overlap with the actual thickness resonance. When evaluating resonance frequency 4 to 5, distinct resonance peaks form next to the predicted resonances, but still do not match properly.

In the second row, the predicted and measured resonances start to coincide. However, there are still multiple small resonances from the eigenmodes. These could still distort the actual position of the thickness resonance, especially when the magnitude is low, e.g., for resonance frequencies 9 and 10.

The third row shows the best match between the predicted and measured resonance frequency. Furthermore, it can be seen that the small peaks between the actual thickness resonances vanished, thus the directivity of the ultrasonic wave must be mainly towards the bottom and no longer to the side. There is electromagnetic interference around 710 kHz and at 900 kHz, as these peaks also occur in the spectrum when there is no mechanical excitation. Based on the measured spectrum, one can now estimate the thickness of the blocks.

## 7. Thickness Estimation

**Figure 14:**
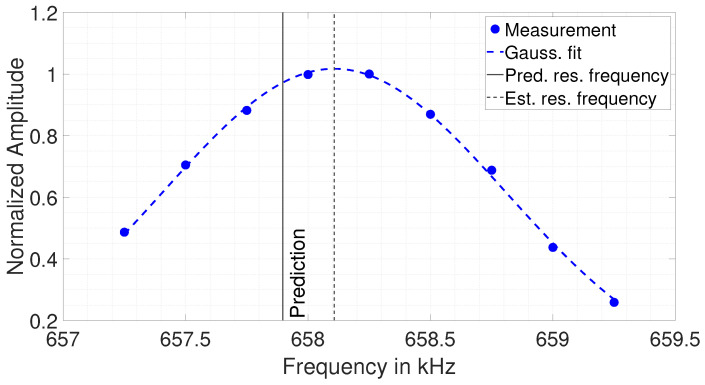
Measured resonance peak at the vicinity of the 11th resonance frequency. From the Gaussian fit, the resonance frequency of the peak is estimated (dashed black line). Also plotted is the predicted resonance frequency (black).

To estimate the thickness of the blocks by the EMAR method, the highest amplitude resonance in the vicinity of the *n*th predicted resonance frequency is searched. Given the found resonance, a Gaussian fit into the resonance peak is performed to estimate the frequency for the maximum of the resonance. An exemplary result of a measured resonance peak (n = 11) with its Gaussian fit is shown in Figure 14.

As can be seen, the Gaussian fit matches the measured resonance peak well. The peak’s frequency is evaluated and used for the thickness estimation. Further, in this figure, the predicted resonance frequency is also plotted. It is clearly visible that the found resonance frequency is shifted compared with the actual predicted resonance frequency. It is interesting to note that all nine measuring points together form a “nice” resonance curve that is perfectly fitted by the Gaussian curve. Therefore, this shift in frequency is not due to an outlier, but must occur from some “systematic” behavior. Probably still the influence of a certain eigenmode, but it could also be some frequency-dependent material behavior affecting the actual shear wave speed compared with the mean velocity of $c_{s}=2401.92\frac{m}{s}$.

The 16 evaluated frequencies based on the position of the maximum of the Gaussian fit for each block are denoted as $f_{\mathrm{res},\mathrm{meas}}$ and serve as input to compute the thickness according to

(11) $$d_{\mathrm{est},n}=n\frac{c}{2f_{\mathrm{res},\mathrm{meas}}}.$$

The method described above is also applied to the simulated EMAR spectrum, shown in Figure 7, and is plotted together with the measurements to compare them.

**Figure 15:**
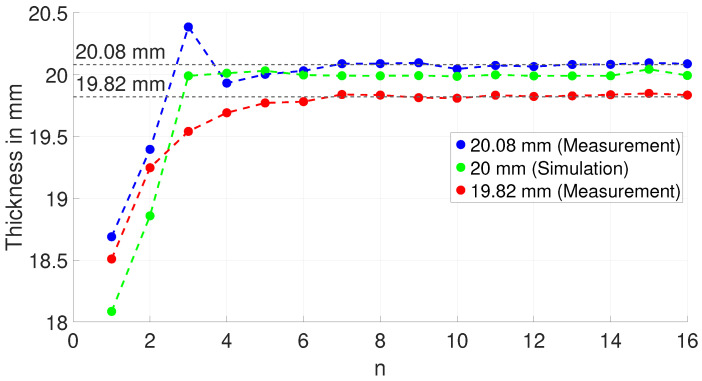
Estimated thickness from *n* evaluated resonance frequencies from measurement and simulation.

The result for the estimated thickness given the evaluated resonance frequencies from the measurements and the simulation is shown in Figure 15.

The measurement shows that the estimated thickness for both blocks significantly differs from the true thickness values for resonance frequencies n < 6. As shown in the simulation results (Figure 8a), this is due to the poor directivity of the excited ultrasonic wave within this frequency range. For higher frequencies (n > 6), the estimated thickness approaches the true value, as the directivity towards the bottom of the block increases. Similar to the measurement, the simulation also approaches the true thickness value of 20 mm once the third or higher frequencies are taken. Most probably, this is due to the fact that the third spatial dimension is missing in the simulation. Therefore, no additional eigenmodes from this third missing dimension interact with the thickness resonances.

**Figure 16:**
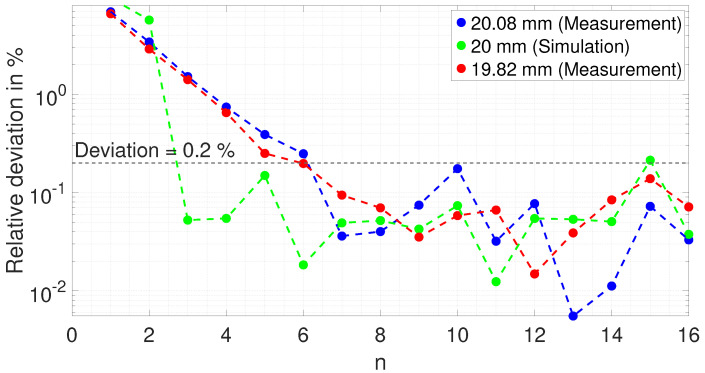
Absolute value of the relative deviation from the true thickness value for the measurements on the 20.08 mm and 19.82 mm copper block and for the simulation of the 20 mm block.

The absolute value of the relative deviation from the true thickness for every resonance frequency is shown in Figure 16.

As with the measurements shown, the relative deviation decreases exponentially from around 8 % to about 0.2 % for resonant frequencies 1 to 6. For higher resonance frequencies, the deviation stays below 0.2 %. The deviation of the simulation shows a similar behavior except that the deviation already remains below 0.2 % for resonance frequencies n ≥ 3.

**Figure 17:**
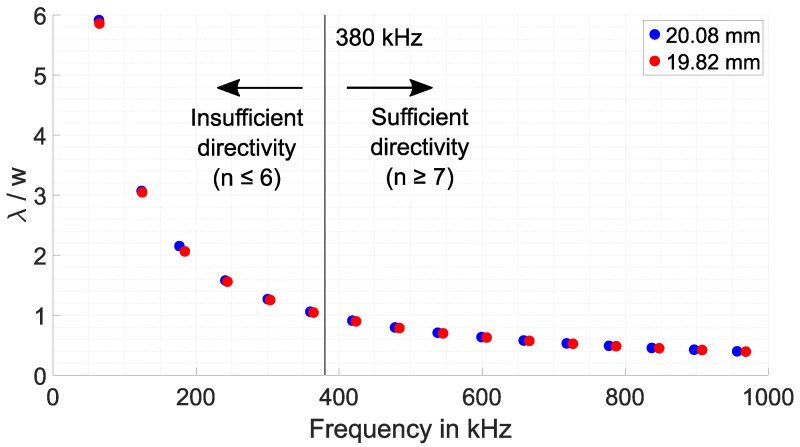
Ratio of wavelength λ over track width w versus the estimated resonance frequencies for the 20.08 mm and 19.82 mm copper block.

Further investigations show that the directivity of the propagating wave and the associated accuracy of the thickness measurement can be related to the EMAT geometry. Figure 17 depicts the ratio between the wavelength λ of the excited wave and EMAT track width w = 6.32 mm versus the estimated resonant frequencies for both blocks.

As can be seen, the ratio decreases with increasing frequency. The ratio is 1 at about 380 kHz. If the ratio is higher, which corresponds to the operation of EMAR at resonant frequencies 1 to 6, the thickness deviation is high due to the weak directivity of the wave, as shown in Figure 16. Increasing the operating frequency (n > 6) and consequently reducing the ratio λ/w to values below 1 increases the directivity, making EMAR suitable for thickness estimation.

## 8. Conclusions

This study demonstrates the successful application of EMAR for thickness measurement on 20 mm thick copper blocks for frequencies in the sub-MHz regime, achieving relative thickness deviations of less than 0.2 %. This shows that even for frequencies below 1 MHz, comparable accuracy to that reported in existing literature at megahertz frequencies can be achieved (see Table 1). Moreover, the operation at lower frequencies enables supply voltages below 60 V, improving safety and reducing equipment costs. In addition to these practical aspects, this work also reveals a critical design parameter not previously discussed in EMAR studies: The ratio of ultrasonic wavelength to EMAT track width (λ/w). We show that for λ/w > 1, wave directivity deteriorates, structural eigenmodes of the specimen become excited, and thickness estimation becomes unreliable. This observation explains why previous works generally operate at much higher frequencies, even when the sample thickness would theoretically allow lower-frequency operation. Overall, this study provides not only a practical low-voltage implementation of EMAR for thick samples, but also establishes a previously unreported design guideline (λ/w < 1) essential for maintaining measurement accuracy. These findings are expected to guide future EMAR system designs, particularly for safe and cost-effective industrial applications where slow wall thinning is monitored over long time periods and inspection speed is less critical.

## Figures and Tables

**Table 2:** Numerical example based on Equation (1) for the first resonance frequency of a 1 mm and 20 mm thick copper sample.

Thickness	Wave Speed	First Resonance (n = 1)
1 mm	2300 m s^−1^ [39]	1.15 MHz
20 mm		58 kHz

**Table 3:** Mechanical parameter range of copper found in the literature and chosen values in this work for the EMAR study.

Parameter	Range	Choice
Young’s Modulus in GPa	110–138	133.5
Density in kg/m^3^	8900–8960	8900
Poisson’s ratio	0.3–0.34	0.3

## Data Availability

The raw data supporting the conclusions of this article will be made available by the authors on request.
